# Prevalence of propionic acidemia in China

**DOI:** 10.1186/s13023-023-02898-w

**Published:** 2023-09-09

**Authors:** Yixing Zhang, Chuwen Peng, Lifang Wang, Sitong Chen, Junwei Wang, Ziheng Tian, Chuangong Wang, Xiaoxin Chen, Suhong Zhu, Guo-Fang Zhang, You Wang

**Affiliations:** 1 School of Clinical Medicine, Jining Medical University, Shandong, 272067 China; 2 School of Basic Medicine, Jining Medical University, 133 Hehua Road, Shandong, 272067 China; 3 Jining Key Laboratory of Pharmacology, Jining Medical University, Shandong, 272067 China; 4 Surgical Research Lab, Department of Surgery, Cooper University Hospital, Camden, NJ 08103 USA; 5 Coriell Institute for Medical Research, Camden, NJ 08103 USA; 6 MD Anderson Cancer Center at Cooper, Camden, NJ 08103 USA; 7 Cooper Medical School of Rowan University, Camden, NJ 08103 USA; 8 Duke Molecular Physiology Institute and Sarah W. Stedman Nutrition and Metabolism Center, Duke University Medical Center, Carmichael Building 48-203, 300 North Duke Street, Durham, NC 27701 USA; 9 Department of Medicine, Division of Endocrinology, Metabolism Nutrition, Duke University Medical Center, Durham, NC 27701 USA

**Keywords:** Propionic acidemia, *PCCA*, *PCCB*, China, Epidemiology, Genotype, Phenotype

## Abstract

Propionic acidemia (PA) is a rare autosomal recessive congenital disease caused by mutations in the *PCCA* or *PCCB* genes. Elevated propionylcarnitine, 2-methylcitric acid (2MCA), propionylglycine, glycine and 3-hydroxypropionate can be used to diagnose PA. Early-onset PA can lead to acute deterioration, metabolic acidosis, and hyperammonemia shortly after birth, which can result in high mortality and disability. Late-onset cases of PA have a more heterogeneous clinical spectra, including growth retardation, intellectual disability, seizures, basal ganglia lesions, pancreatitis, cardiomyopathy, arrhythmias, adaptive immune defects, rhabdomyolysis, optic atrophy, hearing loss, premature ovarian failure, and chronic kidney disease. Timely and accurate diagnosis and appropriate treatment are crucial to saving patients’ lives and improving their prognosis. Recently, the number of reported PA cases in China has increased due to advanced diagnostic techniques and increased research attention. However, an overview of PA prevalence in China is lacking. Therefore, this review provides an overview of recent advances in the pathogenesis, diagnostic strategies, and treatment of PA, including epidemiological data on PA in China. The most frequent variants among Chinese PA patients are c.2002G > A in *PCCA* and c.1301C > T in *PCCB*, which are often associated with severe clinical symptoms. At present, liver transplantation from a living (heterozygous parental) donor is a better option for treating PA in China, especially for those exhibiting a severe metabolic phenotype and/or end-organ dysfunction. However, a comprehensive risk–benefit analysis should be conducted as an integral part of the decision-making process. This review will provide valuable information for the medical care of Chinese patients with PA.

**Supplementary Information:**

The online version contains supplementary material available at 10.1186/s13023-023-02898-w.

## Background

Propionic acidemia (PA) is an autosomal recessive metabolic disorder caused by impaired functioning of propionyl coenzyme A (propionyl-CoA) carboxylase (PCC) in the mitochondria. This results in the metabolic disturbances of propiogenic amino acids (valine, isoleucine, threonine, and methionine), propionate, and odd-chain fatty acids. Patients with PA typically present with vomiting and anorexia, followed by dehydration, weight loss, lethargy, hypothermia, hypotonia, and convulsions. These symptoms can progress rapidly and result in decompensation and poor neurological outcomes. Over time, PA can lead to multiple organ complications, including those affecting the brain, heart, liver, kidney, pancreas, and bone marrow. In severe cases, PA can result in death [1]. Patients with PA typically experience recurrent episodes of hyperammonemia, ketoacidosis, metabolic acidosis, neutropenia, and thrombocytopenia [2]. The clinical presentation is diverse and lacks specificity, with a rapid clinical progression and poor prognosis. Although PA cannot be cured at this time, prompt and proper diagnosis and treatment can stabilize the disease and prevent severe complications.

To gather relevant information for this review, we conducted a literature search from 2010 to 2022 through CNKI and PUBMED databases with the keywords “propionic acidemia”, “organic acidemia”, “metabolic disease”, “newborn screening”, and “China”. Our search yielded a total of 119 papers that met our search criteria and were included in this review.

### Etiology and pathogenesis of PA

#### Structure and function of PCC

PCC is a biotin-dependent carboxylase heterododecamer holoenzyme composed of six α and six β subunits, which is primarily localized in the mitochondria and loosely bound to the inner mitochondrial membrane matrix [3]. Alpha and beta subunits are encoded by the genes *PCCA* (OMIM232000) and *PCCB* (OMIM232050), respectively [4]. *PCCA* gene is located on chromosome 13q32.3 and its encoded α-subunit is 72–80 kDa with three splice isoforms. Classical isoform A (NM_000282.3) is the longest splice isoform encoded by 24 exons containing 728 amino acids. The isoform B is shorter and is encoded by 23 exons containing 702 amino acids. The isoform C is the shortest and is encoded by 23 exons containing 681 amino acids [5]. The *PCCB* gene is located on chromosome 3q22.3 and its encoded β-subunit is 58 kDa. The β-subunit contains 15 exons and encodes 539 amino acids. α-Subunit decorates the outside of a hexamer nucleus formed from 6 β-subunits. α-Subunit contains the N-terminal biotin-binding domain and the C-terminal biotin carboxylase domain, which are responsible for the formation of carboxy biotin after ATP hydrolysis through interaction. β-Subunit hexamer with a propionyl-CoA binding site and a carboxyltransferase domain is responsible for transferring the carboxyl group to propionyl-CoA [6]. Propionyl-CoA is a metabolite that is produced under physiological conditions from propionate, amino acids (valine, isoleucine, threonine, and methionine), side chain of cholesterol, and odd-chain fatty acids. Propionyl-CoA is carboxylated to methylmalonyl-CoA by PCC in the mitochondria and further isomerized to succinyl-CoA. This process is known as the anaplerosis that replenishes the loss of TCA cycle intermediates.

#### Pathogenesis of PA

PA is caused by impaired functioning of PCC due to mutations in the *PCCA* or *PCCB* genes. Mutations of biallelic sites in either *PCCA* or *PCCB* genes or compound heterozygous mutations in two genes result in the loss of PCC activity and lead to abnormal accumulation of propionyl-CoA and its metabolites, such as propionylcarnitine, propionylglycine, 3-hydroxypropionate and 2-methylcitric acid(2MCA). These metabolic disturbances result in a series of biochemical abnormalities and multiple organ complications [7]. Elevated levels of propionyl-CoA can compete with the comparatively lower levels of acetyl-CoA for citrate synthase, leading to the overproduction of 2MCA from propionyl-CoA and oxaloacetate instead of citrate. This excessive 2MCA production in propionic acidemia diverts oxaloacetate away from the tricarboxylic acid (TCA) cycle. 2MCA is reported to be an inhibitor of multiple enzymes involved in TCA cycle, such as citrate synthase, aconitase, isocitrate dehydrogenase, in rat liver mitochondria [8]. The harmful effects on mitochondria induced by 2MCA may play a role in the development of brain damage and neurological complications in patients with PA [9]. High levels of propionyl-CoA and its metabolites are reported to inhibit multiple enzymes including pyruvate dehydrogenase complex and respiratory chain complexes in the TCA cycle, hindering mitochondrial energy production [10]. Additionally, the production of succinyl-CoA from propionyl-CoA is also reduced. The above metabolic perturbations could inhibit TCA cycle flux and impair energy production. Life-threatening acute metabolic decompensations (AMD) are a prominent feature in PA, characterized biochemically by hyperammonemia, metabolic acidosis with a high anion gap, and lactic acidosis [11]. Hyperammonemia is primarily caused by urea cycle disorders. Propionyl-CoA acts as a competitive inhibitor of N-acetylglutamate synthase, which reduces the synthesis of N-acetylglutamate. N-acetylglutamate is an agonist of carbamylphosphate synthase-1, and its reduction can subsequently lead to a decrease in the activity of carbamylphosphate synthase-1 in the urea cycle [12]. Some evidence also suggests the amino acid substrates of the urea cycle, especially citrulline, ornithine and arginine are reduced in patients with PA [13]. During PA decompensations, the body's compensatory mechanisms rely heavily on glutamate/glutamine metabolism to convert α-ketoglutarate. This process results in an excessive generation of ammonia. Another potential mechanism to explain the occurrence of hyperammonemia during PA decompensations is the decrease in glutamine levels [14].

Significantly higher concentrations of the branched-chain amino acids (BCAAs), including leucine (Leu), valine (Val), and isoleucine (Ile), and their intermediate metabolites have been observed during decompensation episodes. This suggests that there may be an increase in protein catabolism. The breakdown of BCAAs acutely increases the amount of circulating toxic metabolites. Due to their acidic nature, these metabolites can rapidly lead to metabolic acidosis by decreasing the pool of bicarbonate in the body [9, 15]. Lactate acidosis may result from the decline of pyruvate dehydrogenase activity, which can lead to the excessive conversion of pyruvate to lactate. Elevated levels of propionate and its metabolites 3-hydroxypropionate and methylcitrate can cause bone marrow suppression, which may lead to anemia, granulocytopenia, and thrombocytopenia [16]. In addition, severe anemia in PA could be ascribed to low levels of BCAA in plasma, which could be a result of a low natural protein intake and high demands for protein synthesis [17]. Ketoacidosis is often reported in PA with metabolic decompensation and its underlying mechanism remains unclear. Studies on the PA mouse model have suggested that propionate overload can stimulate ketone production by increasing fatty acid oxidation in the liver via the lowering of malonyl-CoA [18]. Furthermore, the accumulation of propionyl-CoA can interfere with glycine cleavage by reducing H protein production, leading to hyperglycinemia [19]. Hepatic encephalopathy resulting from hyperammonemia may be a major cause of neurological damage in individuals with PA.

### Clinical features and diagnosis

Patients with PA exhibit a diverse range of clinical manifestations that can present from infancy to adulthood. These manifestations are typically categorized as either early-onset (occurring at or before 3 months of age) or late-onset (occurring after 3 months of age) [20]. Early-onset typically affects newborns who were born with normal gestation and delivery. Patients may not exhibit symptoms immediately after birth and can remain asymptomatic for hours, days, or even months. However, they are at high risk of sudden acute metabolic decompensation, which can be life-threatening. Children with early-onset PA often exhibit symptoms such as poor feeding, lethargy, disorders of consciousness, repeated vomiting, convulsions, dyspnea, growth retardation, epilepsy and motor disorders. Late-onset PA can be divided into two types: chronically progressive and intermittent seizure. The intermittent onset type has an acute phase followed by a stable phase. The acute decompensation phase is typically triggered by metabolic stress, such as infection, prolonged or intense physical exercise, injury, surgery and/or general anesthesia, excessive protein intake, and the attack has a neonatal-like onset. In contrast, the stable phase can be associated with various complications [21].

The most common complications associated with PA are cardiac and neurological disorders [22]. Cardiac complications include cardiomyopathy and arrhythmias. Chronic neurological and cognitive complications are frequent in PA, including movement disorders, spastic paresis, intellectual disability and strokes of basal ganglia [23]. Late manifestations of neuropsychological disorders, such as autism and borderline personality traits, have also been reported [24, 25]. Other complications may include recurrent pancreatitis, adaptive immune defects, rhabdomyolysis, optic atrophy, hearing loss, premature ovarian failure, and chronic kidney disease [26–29]. In China, most of the reported clinical cases are early-onset. Delayed cardiomyopathy and neurological complications have also been reported in individual cases. The characteristics and clinical data of patients we reviwed are presented in Table 3.

Neonatal screening by mass spectrometry is currently an effective tool for early identification and diagnosis of PA. The diagnosis of PA is confirmed by measuring the levels of C3 and the ratio of C3/acetylcarnitine (C2) in blood by liquid chromatography-tandem mass spectrometry (LC–MS/MS). The typical range of values for C3 (propionylcarnitine) is between 0.2 and 4.3 μmol/L. Meanwhile, the C3/C2 (acetylcarnitine) ratio typically falls between 0.03 and 0.2 [61]. In addition, the levels of 3-hydroxypropiona, propionylglycine and 2MCA in urine can also be measured using gas chromatography-mass spectrometry (GC–MS) to aid in diagnosis [20]. When PA is suspected, further examinations should be conducted to confirm the initial biochemical diagnosis. General tests such as blood ammonia, blood glucose, blood gas analysis, and myocardial zymogram analysis, as well as urine tests for ketone bodies and organic acids, can be conducted to aid in diagnosis [30]. In China, patients with early-onset PA are often misdiagnosed with intermittent neurological deterioration and neonatal sepsis [16]. Following standard clinical and analytical procedures is critical for the differential diagnosis. In newborns with clinical distress and suspicion of sepsis, seizures, or organic acidemias must be considered in the differential diagnosis from the outset [21, 22]. PA and methylmalonic acidemia (MMA) are both disorders in the propionyl-CoA metabolic pathway with shared clinical presentations. PA and MMA are not easily distinguished when screening for inherited metabolic disorders in newborns. Both MMA and PA can cause an elevation of C3 in the acylcarnitine spectrum, which is a common metabolic biomarker for both disorders. However, since PA directly causes an increase in C3, the rise of C3 is usually more pronounced with PA than with MMA in theory, as demonstrated in clinical cases [31, 32]. Determination of methylmalonate from dried blood spots (DBS), serum, and urine samples is commonly performed for differential diagnosis of PA and MMA in clinical diagnostics and newborn screening. The biochemical indicators of PA include elevated 2MCA and propionylglycine, and C3, while high urinary methylmalonate is specific to MMA [33]. Higher levels of 2MCA are generally observed in patients with PA than with MMA. Additionally, PA patients with a severe phenotype and significant long-term complications tend to have even higher levels of 2MCA [34]. A retrospective study conducted at a single center revealed that C3 level, C3/C2 ratio, and 2MCA level in the amniotic fluid supernatant are reliable biochemical markers for the diagnosis of PA. This study also suggested that the C3/C2 ratio is the most dependable biochemical marker for the prenatal diagnosis of PA [35]. Table 1 summarizes the various biomarkers used for the differential diagnosis of PA and MMA and can serve as a reference for clinical practice. The diagnosis of PA is confirmed through the analysis of mutations in the *PCCA* and *PCCB* genes, as well as the measurement of PCC enzyme activity. Sanger sequencing, quantitative polymerase chain reaction (qPCR), next generation sequencing (NGS) are employed to detect and identify the pathogenic mutations of biallelic sites in *PCCA* or *PCCB* genes. In special cases, additional technologies such as cDNA analysis, multiplex ligation-dependent probe amplification (MLPA), or long-read whole-genome sequencing may be necessary to improve the detection rates [36]. Splicing variants need to be confirmed at the mRNA level. The identification of biochemical markers and gene mutations can aid in family genetic counseling and prenatal diagnosis, particularly for families with a proband affected by PA [37].

**Table 1:** Biochemical biomarker of PA and MMA

Potential biomarkers	PA	MMA
Organic acids		
2‐methylcitric acid	↑	↑
Methylmalonic acid	_	↑↑
Propionic acid	↑↑	↑
3‐hydroxypropionic acid	↑	↑
Conjugates		
Propionylglycine	↑	↑
Ketonuria	↑↑	↑
Carnitine panel		
Acetylcarnitine (C2)	↓	↓
Propionylcarnitine (C3) and C3/C2 ratio	↑↑	↑↑
Methylmalonylcarnitine	_	↑↑
Acyl‐CoAs		
Propionyl‐CoA	↑↑	↑
Methylmalonyl‐CoA	↑	↑↑
Odd‐numbered long‐chain fatty acids	↑	↑
Acetyl‐CoA	↓	↓
Tricyclic acid (TCA) cycle intermediates		
Citric	↓	↓
Ketoglutaric	↓	↓
Succinic	↓	↓
Malic acid	↓	↓
Ammonium	↑	↑
Lactic acid	↑	↑
Amino acids		
Alanine:Serine	↑	↑
Alanine:Lysine	↓	↓
Glycine	↑	↓

### Epidemiology data of PA in China

The incidence of PA varies across different countries and regions, with global estimates ranging from 1/313,000 to 1/1000. In the United States, the estimated live-birth incidence is 1/105,000–1/130,000 [32, 38] and in Italy 1/166,000 [39]. In a selective and expanded newborn screening, the frequencies of PA in Japan, South Korea, and Germany were 1/41,000, 1/313,000 and 1/250,000, respectively [40]. The birth incidence in the Middle East is generally higher, with rates of 1/20,000–1/45,000 in the United Arab Emirates [41] and 1/28,000 in Saudi Arabia [42]. Some Saudi tribes have even higher rates, ranging from 1/2000 to 1/5000 [43]. The highest birth incidence (1/1000) is among the Greenlandic Inuits [44].

**Fig. 1:**
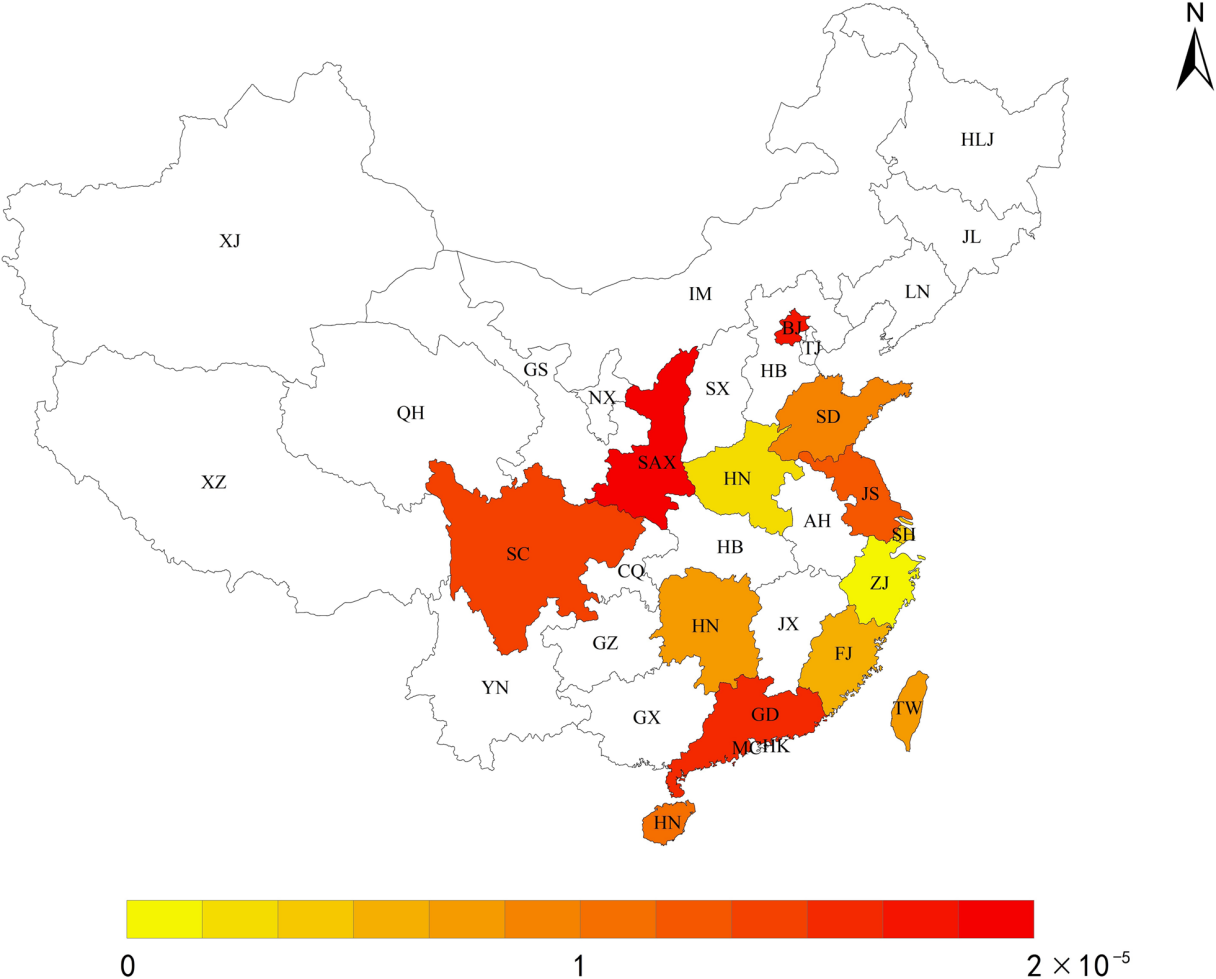
Distribution and incidence of PA in each province/municipality in China. HLJ, Heilongjiang; JL, Jilin; LN, Liaoning; XJ, Xinjiang; IM, Inner Mongolia; BJ, Beijing; TJ, Tianjin, HEB, Hebei; SX, Shanxi; SAX, Shaanxi; NX, Ningxia; GS, Gansu; QH, Qinghai; SD, Shandong; JS, Jiangsu; AH, Anhui; HEN, Henan; SH, Shanghai; HUB, Hubei; CQ, Chongqing; SC, Sichuan; ZJ, Zhejiang; JX, Jiangxi; HUN, Hunan; GZ, Guizhou; YN, Yunnan; FJ, Fujian; TW, Taiwan; GD, Guangdong; GX, Guangxi; HN, Hainan, HK, Hongkong; MC, Macau. Areas without color indicate that those without screening data. The darker the color the higher the incidence

In mainland China, newborn screening by mass spectrometry was first introduced in 2004 [45]. With the wide application of LC–MS/MS and GC/MS, newborn screening for PA has been available and reported in most parts of China. However, the overall prevalence of PA in China remains unknown. We conducted a retrospective study analyzing screening data from different districts of China and collected incidence data of neonatal inherited metabolic diseases from 23 provinces or municipalities over the last decade (from 2010 to 2022). The results are presented in Table 2. Furthermore, we mapped out the provincial-level prevalence of PA in China in Fig. 1, based on the reported cases.

**Table 2:** Epidemiological reports of PA patients in China

Province/municipality	Time Period	Screened newborns	Confirmed PA	Incidences	References
North China					
Beijing	From January 1st, 2009 to July 2009	11,240	1	1/11,240	[96]
Shandong		673,102	9	1/74,789	
Jining	From July 2014 to December 2018	51,4234	8	1/64,279	[67]
Qingdao	From January 2012 to December 2016	158,868	1	1/158,868	[53]
Henan	From January 2013 to August 2019	850,486	3	1/283,495	[48]
Kaifeng	From August 2015 to September 2017	91,406	1	1/91,406	[97]
Jiangsu		718,258	15	1/47,884	
Xuzhou	From September 2015 to September 2018	297,610	8	1/37,201	[61]
Yancheng	From 2012 to 2014	18,988	6	1/3,165	[49]
Shaanxi	From January 2013 to October 2014	10,205	2	1/5,103	[98]
South China					
Shanghai	From 2010 to 2016	760,000	2	1/200,000	[99]
Zhejiang	From January 2009 to December 2016	1,861,262	6	1/310,200	[51]
Wenzhou	From October 2013 to December 2018	489,148	1	1/489,148	[47]
Fujian		364,545	2	1/182,273	
Quanzhou	From January 2014 to November 2018	364,545	2	1/182,273	[100]
Hunan	From March 2013 to September 2017	565,182	4	1/141,296	[101]
Huaihua	From March 2015 to December 2017	79,205	1	1/79,205	[102]
Guangdong		577,037	22	1/26,229	
Foshan	From August 1st, 2002 to March 30th, 2007	11,087	1	1/11,087	[103]
Guangning	From October 2010 to October 2015	8,238	16	1/515	[46]
Puning	From January 2007 to December 2011	115,219	2	1/57,610	[104]
Huizhou	From May 2012 to May 2013	123,231	1	1/123,231	[105]
Guangzhou	From January 2015 to December 2020	272,117	1	1/272,117	[106]
Meizhou	From April 2019 to July 2021	47,145	1	1/47,145	[107]
Taiwan	From 2001 to 2014	1,390,000	3	1:464,000	[40]
Sichuan	From November 2017 to December 2018	39,648	1	1/39,648	[52]
Hainan	From January 2016 to December 2019	54,506	1	1/54,506	[108]
Jiangsu					
Suzhou	From 2014 to October 2019	401,660	1	1/401,660	[109]

To better analyze the incidence of PA in China, we divided country into two regions based on the Qinling Mountains-Huaihe River line, namely the northern and southern regions. The overall incidence of PA in the southern region of China was found to be higher, ranging from 1/489,148 to 1/515, with the highest in Guangning city, Guangdong Province. Out of 8,238 neonates screened from October 2010 to October 2015 in Guangning, 16 were diagnosed with PA [46]. The lowest incidence was found in Wenzhou city, Zhejiang Province, where 489,148 newborns were screened from October 1, 2013 to December 31, 2018, and the incidence of PA was reported as 1/489,148 [47].

The overall incidence rate of PA in northern China was from 1/283,459 to 1/3,156. Among 850,486 neonates screened in Henan province from January 2013 to August 2019 [48], three cases of PA were detected with an incidence rate of 1/283,459, which was the lowest in northern China. On the other hand, in Yancheng city, Jiangsu Province, 6 cases of PA were diagnosed among 18,988 screened neonates from June 2012 to June 2014, resulting in an incidence rate of 1/3,156, which was the highest in northern China [49].

Our survey included 15 regions in the south and 8 regions in the north based on available reports. However, cases of PA reported in other regions of China without regional incidence data were not included in this review. For example, from 2007 to 2010, 113 cases of PA were diagnosed from 5,931 children screened in major hospitals in Hebei province. Additionally, PA is a common organic acidemia found in Shijiazhuang [50]. Table 2. displays the epidemiological data of PA in China, indicating a significant regional difference in PA incidence across the country. The variation in incidence might be due to differences in genetic and ethnic backgrounds, as well as the total number of people screened. Moreover, the dates of newborn screening differ among various regions. For instance, in Zhejiang Province, the number of newborns screened for PA reached 1,861,262 from January 2009 to December 2016 [51], while the screening data of Sichuan Province was only collected from November 2017 to December 2018 and the number of newborns screened was 39,648 in this area [52]. In addition, various regions have different newborn screening rates conducted at disparate times. For example, Qingdao reported a screening rate of 79.0% in 2016 [53], while Yancheng reported a screening rate of 29.0% from January to December in 2012 [49].

### Correlation between genotypes and phenotypes of PA in China

The correlation between gene mutations and clinical phenotype in PA patients has become a hot topic due to the increase in reported PA cases in recent years. To contribute to this field, we conducted a study analyzing the PCC mutations in 61 Chinese patients with PA reported in literature since 2014 and examined the phenotype-genotype correlations.

#### *PCCA* and *PCCB* variants in Chinese PA patients

Among 61 patients, 25 (41.0%) patients harbored *PCCA* variants including three pairs of siblings and 36 (59.0%) harbored *PCCB* variants including two pairs of siblings. There were 48 variants in *PCCA* and 66 variants in *PCCB*. Seventeen (27.9%) patients were diagnosed with newborn screening or prenatal diagnosis. Of the remaining 49 patients, 35 had an early-onset (≤ 3 months) disease and 7 had a late-onset (> 3 months) disease. Following the guidelines developed by the American College of Medical Genetics and Genomics (ACMG), we classified the gene mutations into five levels: pathogenic, likely pathogenic, benign, likely benign, and uncertain significance. We also listed the CADD pathogenicity score, and the mutation significance cut-off score was set at 20 (Table 3.).

**Table 3:** Gene mutations found in 61 Chinese patients with PA

Number	Exon/Intron	Nucleotide alteration	Protein alteration	Mutation type	Early/late onset	ACMG classification	CADD scores	MSC scores	References	Clinical features	Treatment	Outcome
*PCCA*												
1	Exon10	NM_000282.4:c.802C > T	p.Arg268Cys	Missense	Late	Pathogenic	22.5	20	[110]	No specific clinical symptoms	NA	Following up eleven times within one year and three months after birth, liver and kidney function were normal
	Exon11	NM_000282.4:c.827delG	p.Gly276Valfs*46	Frameshift		Likely Pathogenic	NA	NA				
2	Intron8	NM_000282.4:c.638-1G > C	NA	Splice site mutation	Early	Likely Pathogenic	33	20	[61]	Mild anemia or mild jaundice, hyperammonemia, hyperlactatemia and hypoglycemia	Protein restriction with special protein powder and sufficient calories, L-carnitine and arginine supplementation, and regular follow-up checks	Normal development and no acute metabolic disorders were observed during the course of management
	NA	NA	NA	NA		NA	NA	NA				
3	Exon9	NM_000282.4:c.688C > T	p.Arg230Cys	Missense		Pathogenic	28.3	20				
	Intron13	NM_000282.4:c.1209 + 2 T > G	NA	Splice donor		Pathogenic	34	20				
4	Intron2	NM_000282.4:c.183 + 1G > C	NA	Splice site mutation		Likely Pathogenic	33	20				
	Exon21	NM_000282.4:c.1850 T > C	p.Leu617Pro	Missense		Likely Benign	24.5	20				
5	Exon22	NM_000282.4:c.2002G > A	p.Gly668Arg	Missense		Pathogenic	32	20				Development delay in early infancy
	Exon22	NM_000282.4:c.2040G > A	p.Ala680Ala	Synonymous		Likely Pathogenic	24	20				
6	Exon22	NM_000282.4:c.2002G > A	p.Gly668Arg	Missense		Pathogenic	32	20				
	Exon2	NM_000282.4:c.131delinsATT	p.Cys44Tyrfs*3	Missense		Pathogenic	NA	NA				
7	Intron3	NM_000282.4:c.231 + 1G > A	NA	Splice site mutation		Likely Pathogenic	34	20				NA
	Exon7	NM_000282.4:c.596 T > A	p.Val199Asp	Missense		Uncertain Significance	28.1	20				
8	Exon22	NM_000282.4:c.2002G > A	p.Gly668Arg	Missense	Early	Pathogenic	32	20	[59]	Respiratory failure, granulocytopenia, and cardiac damage with rapid progression	Anti-infection measures, atomization therapy, sputum suction, glucose infusion, protein restriction, and supplementation with calcium, arginine, l-carnitine, and l-creatinine phosphate. Efforts were also made to strengthen the respiratory tract and correct acidosis	Died at the age of eighth day after birth
	Exon22	NM_000282.4:c.2002G > A	p.Gly668Arg	Missense		Pathogenic	32	20				
9	Exon22	NM_000282.4:c.2002G > A	p.Gly668Arg	Missense		Pathogenic	32	20		Disorders of consciousness, poor feeding, hyperammonemia, and metabolic acidosis	Anti-infection, fasting, restriction of protein intake, supplementation of hypertonic glucose, arginine iv to promote ammonia excretion	Died at the age of one month and seven days after birth
	Exon22	NM_000282.4:c.2002G > A	p.Gly668Arg	Missense		Pathogenic	32	20				
10	Intron20	NM_000282.4:c.1845 + 1G > A	NA	Splice site mutation	Early	Pathogenic	34	20	[111]	NA	NA	Died during the neonatal period
	Exon6	NM_000282.4:c.446del	p.Asn149Thrfs*35	Frameshift		Pathogenic	NA	NA				
11	Intron20	NM_000282.4:c.1845 + 1G > A	NA	Splice site mutation	NA	Pathogenic	34	20		Coma, convulsions, bronchopneumonia, anemia, thrombocytopenia	NA	Died after more than seven months old
	Exon6	NM_000282.4:c.446del	p.Asn149Thrfs*35	Frameshift		Pathogenic	NA	NA				
12	Exon3	NM_000282.4:c.229C > T	p.Arg77Trp	Missense	NA	Pathogenic	25.1	20	[112]	NA	NA	NA
	Intron21	NM_000282.4:c.1899 + 1G > A	NA	Splice site mutation		Likely Pathogenic	NA	NA				
13	Exon14	NM_000282.4:c.1262A > C	p.Gln421Pro	Missense	Newborn screening	Uncertain Significance	22.8	20	[64]	No typical PA symptoms, no hyperammonemia	Low-protein diet and l-carnitine supplementation	No significant development delay
	Exon3	NM_000282.4:c.229C > T	p.Arg77Trp	Missense		Pathogenic	25.1	20				
14	Exon15	NM_000282.4:c.1288C > T	p.Arg430Ter	Nonsense	Newborn screening	Pathogenic	37	20	[113]	NA	NA	NA
	Exon3	NM_000282.4:c.229C > T	p.Arg77Trp	Missense		Pathogenic	25.1	20				
15	Intron13	NM_000282.4:c.1210-7C > G	NA	Splice site mutation	Newborn screening	Uncertain Significance	25.3	20		NA	NA	NA
	Exon13	NM_000282.4:c.1185A > C	p.Ala395Ala	Samesense		Likely Benign	9.037	20				
16	Exon22	NM_000282.4:c.2002G > A	p.Gly668Arg	Missense	Newborn screening	Pathogenic	32	20	[114]	NA	NA	NA
	Exon22	NM_000282.4:c.2002G > A	p.Gly668Arg	Missense		Pathogenic	32	20				
17	Exon12	NM_000282.4:c.937C > T	p.Arg313Ter	Nonsense	Early	Pathogenic	38	20	[37]	Tachypnea, poor reaction, seizures, lethargy, irritability	Antibiotics treatment, supplementation of l-carnitine, folic acid, and biotin; protein restriction	Died at approximately two months of age
	Exon10-	NM_000282.4:c.773_819 + 47delinsAA	NA	Complex deletion–		Likely Pathogenic	NA	NA				
	Intron10			Insertion (delins) mutation								
18	Exon15	NM_000282.4:c.1288C > T	p.Arg430Ter	Nonsense	Early	Pathogenic	37	20	[7]	Generalized tonic–clonic seizures, metabolic acidosis and hyperammonemia	NA	Died at six and a half months old from sudden cardiac arrest
	Exon22	NM_000282.4:c.2002G > A	p.Gly668Arg	Missense		Pathogenic	32	20				
19	Exon16	NM_000282.4:c.1426C > T	p.Arg476Ter	Nonsense	Early	Pathogenic	41	20		Recurrent vomiting, lethargy and dyspnea	Low-isoleucine, − methionine, − threonine, and-valine diet, supplementation of l-carnitine and biotin	Mild intellectual disability
	Exon16	NM_000282.4:c.1426C > T	p.Arg476Ter	Nonsense		Pathogenic	41	20				
20	Exon19	NM_000282.4:c.1746G > C	P.Ser582Ser	Synonymous	Early	Uncertain Significance	9.081	20	[36]	Cough, tachypnea, dyspnea and metabolic acidosis	NA	Died due to respiratory failure
	Exon3-4	NM_000282.4:Exon3-4del	NA	NA		NA	NA	NA				
21	Exon19	NM_000282.4:c.1746G > C	P.Ser582Ser	Synonymous	Early	Uncertain Significance	9.081	20		Cough, tachypnea, dyspnea and metabolic acidosis	NA	NA
	Exon3-4	NM_000282.4:Exon3-4del	NA	NA		NA	NA	NA				
22	Exon22	NM_000282.4:c.2002G > A	p.Gly668Arg	Missense	Late	Pathogenic	32	20	[62]	Poor feeding, intermittent vomiting, dilated cardiomyopathy	Liver transplantation	Liver transplantation improved cardiac function but did not significantly impact growth, even with a normal diet, except when supplemented with l-carnitine
	Exon22	NM_000282.4:c.2002G > A	p.Gly668Arg	Missense		Pathogenic	32	20				
23	Exon21	NM_000282.4:c.1850 T > C	p.Leu617Pro	Missense	Newborn screening	Likely Benign	24.5	20	[108]	NA	NA	NA
	Exon4	NM_000282.4:c.297 T > A	p.Ser99Arg	Missense		Uncertain Significance	20.4	20				
24	Intron15	NM_000282.4:c.1353 + 5_1353 + 9del	NA	Splice site mutation	Late	Uncertain Significance	NA	NA	[52]	NA	Low protein diet, special milk powder, oral l-carnitine and arginine	Physical and intellectual development is normal and noacute metabolic disorders
	NA	NA	NA	NA		NA	NA	NA				
25	Exon3	NM_000282.4:c.229C > T	p.Arg77Trp	Missense	Newborn screening	Pathogenic	25.1	20	[109]	NA	NA	NA
	Exon22	NM_000282.4:c.2002G > A	p.Gly668Arg	Missense		Pathogenic	32	20				
*PCCB*												
1	Exon13	NM_000532.5:c.1301C > T	p.Ala434Val	Missense	Early	Pathogenic	32	20	[65]	Poor feeding, hyperglycinemia, hyperammonemia, metabolic acidosis, early recurrent infections, and development delay	Sodium bicarbonate IV to correct acidosis, l-carnitine supplementation, BCAA restriction and protein intake reduction	Obvious development delay and intellectual disability
	Exon13	NM_000532.5:c.1301C > T	p.Ala434Val	Missense		Pathogenic	32	20				
2	Exon1	NM_000532.5:c.167_179del13insC	p.Asp56_Lys60delinsAla	Deletion and insertion mutation	Late	NA	NA	NA		Hyperglycininemia	l-carnitine supplementation, BCAA restriction and protein intake reduction	Mental and language development is slightly delayed
	Exon1	NM_000532.5:c.167_179del13insC	p.Asp56_Lys60delinsAla	Deletion and insertion mutation		NA	NA	NA				
3	Exon1	NM_000532.5:c.132_134delGACinsAT	p.Thr45SerfsTer20	Deletion and insertion mutation	Early	Likely Pathogenic	NA	NA	[115]	Repeated seizures, hyperammonemia, ketoacidosis, hyperglycaemia, anemia	Protein restriction along with phenobarbital, l-carnitine, and arginine supplementation. Treatments to correct acidosis and electrolyte disorders	Symptoms were improved
	NA	NA	NA	NA		NA	NA	NA				
4	Exon14	NM_000532.5:c.1403C > T	p.Ala468Val	Missense	Early	Likely Pathogenic	27.1	20	[61]	Mild anemia or mild jaundice, metabolic acidosis, hyperammonemia, hyperlactatemia and hypoglycemia	Similar to PCCA cases No. 2- 7	Physical and intellectual development is normal
	Exon8	NM_000532.5:c.838dup	p.Leu280fs	Frameshift		Pathogenic	NA	NA				
5	Intron1	NM_000532.5:c.184-2A > G	NA	Splice site mutation	Early	Pathogenic	33	20		Repeated vomiting, lethargy, dyspnea, hypotonia, metabolic acidosis, hyperammonemia, liver dysfunction		Death from an acute metabolic disorder occurred at the age of seven months
	Exon7	NM_000532.5:c.733G > A	p.Gly245Ser	Missense		Likely Pathogenic	28.9	20				
6	Exon3	NM_000532.5:c.331C > T	p.Arg111Ter	Nonsense	Early	Pathogenic	36	20	[116]	Lethargy, poor feeding	l-carnitine, special milk powder	Died at three months old after birth
	Exon12	NM_000532.5:c.1228C > T	p.Arg410Trp	Missense		Pathogenic	37	20				
7	Exon1	NM_000532.5:c.146delG	p.Gly49Glufs*16	Frameshift	Late	Likely Pathogenic	NA	NA		Vomiting, lethargy, poor spirit	l-carnitine, special milk powder	Poor compliance and intermittent treatment, significant development delay
	Exon12	NM_000532.5:c.1253C > T	p.Ala418Val	Missense		Likely Pathogenic	27.4	20				
8	Exon10	NM_000532.5:c.1087 T > C	p.Ser363Pro	Missense	Late	Likely Pathogenic	30	20	[47]	NA	NA	NA
	Exon10	NM_000532.5:c.1087 T > C	p.Ser363Pro	Missense		Likely Pathogenic	30	20				
9	Intron1	NM_000532.5:c.184-2A > G	NA	Splice site mutation	Early	Pathogenic	33	20	[117]	Abdominal distension, vomiting, poor feeding, dyspnea, and hyperammonemia	NA	NA
	Exon7	NM_000532.5:c.733G > A	p.Gly245Ser	Missense		Likely Pathogenic	28.9	20				
10	NA	NA	NA	NA	Late	NA	NA	NA	[64]	Recurrent vomiting, disorders of consciousness, hyperventilation, and metabolic acidosis	NA	Died at the age of two years and eight months after birth
	NA	NA	NA	NA		NA	NA	NA				
11	Exon13	NM_000532.5:c.1316A > G	p.Tyr439Cys	Missense	Prenatal diagnosis	Pathogenic	31	20		NA	NA	Miscarriage occurred at twenty-one weeks of gestation
	Intron1	NM_000532.5:c.-4156_183 + 3713del	NA	NA		NA	NA	NA				
12	Exon13	NM_000532.5:c.1301C > T	p.Ala434Val	Missense	Early	Pathogenic	32	20		Jaundice, poor feeding, hypotonia, metabolic acidosis, hyperglycaemia, and hyperammonemia	NA	NA
	Exon5	NM_000532.5:c.580 T > C	p.Ser194Pro	Missense		Likely Pathogenic	28.8	20				
13	Exon13	NM_000532.5:c.1301C > T	p.Ala434Val	Missense	Early	Pathogenic	32	20		Poor feeding, severe jaundice, metabolic acidosis, and hyperammonemia	NA	Died at the age of one year and eight months after birth
	Exon13	NM_000532.5:c.1301C > T	p.Ala434Val	Missense		Pathogenic	32	20				
14	NA	NA	NA	NA	Early	NA	NA	NA		Poor feeding, vomiting, hyperammonemia, metabolic acidosis, and recurrent infections	NA	Died at the age of one year and six months of a severe infection
	NA	NA	NA	NA		NA	NA	NA				
15	Intron1	NM_000532.5:c.-4156_183 + 3713del	NA	NA	Prenatal diagnosis	NA	NA	NA		Poor feeding, vomiting, and hyperammonemia	l-carnitine, special milk powder, phenylbutyric acids	Moderate developmental delay
	Exon13	NM_000532.5:c.1301C > T	p.Ala434Val	Missense		Pathogenic	32	20				
16	Exon13	NM_000532.5:c.1301C > T	p.Ala434Val	Missense	Newborn screening	Pathogenic	32	20		Classic PA phenotype and moderate developmental delay	Low-protein diet supplemented with l-carnitine, metronidazole, and growth hormone, liver transplantation	No classical PA phenotypic symptoms was observed during the twelve-month follow-up after liver transplantation
	Exon15	NM_000532.5:c.1534C > T	p.Arg512Cys	Missense		Pathogenic	28.6	20				
17	Exon13	NM_000532.5:c.1301C > T	p.Ala434Val	Missense	Early	Pathogenic	32	20		Hypoactivity, poor feeding and tachypnea hypotonia, hepatomegaly, disorders of consciousness, and hyperammonemia	NA	Died at six days old after birth
	Exon8	NM_000532.5:c.838dup	p.Leu280fs	Frameshift		Pathogenic	NA	NA				
18	Intron1	NM_000532.5:c.-4156_183 + 3713del	NA	NA	Newborn screening	NA	NA	NA		Hyperammonemia and hypoglycemia	Low-protein diet supplemented with l-carnitine	Mild development delay
	Exon13	NM_000532.5:c.1301C > T	p.Ala434Val	Missense		Pathogenic	32	20				
19	Exon8	NM_000532.5:c.838dup	p.Leu280fs	Frameshift	Newborn screening	Pathogenic	NA	NA	[67]	NA	NA	NA
	Exon13	NM_000532.5:c.1316A > G	p.Tyr439Cys	Missense		Pathogenic	31	20				
20	Exon8	NM_000532.5:c.838dup	p.Leu280fs	Frameshift		Pathogenic	NA	NA				
	Exon13	NM_000532.5:c.1316A > G	p.Tyr439Cys	Missense		Pathogenic	31	20				
21	Exon3	NM_000532.5:c.370C > T	p.Gln124Ter	Nonsense		Likely Pathogenic	48	20				
	Exon12	NM_000532.5:c.1283C > T	p.Thr428Ile	Missense		Pathogenic	27.9	20				
22	Exon3	NM_000532.5:c.331C > T	p.Arg111Ter	Nonsense		Pathogenic	36	20				
	Exon10	NM_000532.5:c.1087 T > C	p.Ser363Pro	Missense		Likely Pathogenic	30	20				
23	Exon12	NM_000532.5:c.1220del	p.Gly407Alafs*36	Frameshift		Pathogenic	NA	NA				
	Exon10	NM_000532.5:c.1015A > T	p.Asn339Asp	Missense		Likely Pathogenic	29.3	20				
24	Exon13	NM_000532.5:c.1316A > G	p.Tyr439Cys	Missense	Newborn screening	Pathogenic	31	20				
	NA	NA	NA	NA		NA	NA	NA				
25	Exon8	NM_000532.5:c.838dup	p.Leu280fs	Frameshift	Early	Pathogenic	NA	NA	[16]	Hypotonia, disorders of consciousness, pancytopenia metabolic acidosis and hyperammonemia	Protein restriction and supplementation of l-carnitine, mannitol, and calories. Correct acidosis and electrolyte disorders	Died from pneumonia approximately thirty days after birth
	Exon8	NM_000532.5:c.838dup	p.Leu280fs	Frameshift		Pathogenic	NA	NA				
26	Exon6	NM_000532.5:c.634G > T	p.Asp212Tyr	Missense	Early	Likely Pathogenic	29.6	20	[118]	Poor feeding, hypotonia, pancytopenia, hyperlactatemia	l-carnitine, vitamin B12, special milk powder, a restricted protein diet	Development delay, intellectual disability
	Exon8	NM_000532.5:c.838dup	p.Leu280fs	Frameshift		Pathogenic	NA	NA				
27	Exon3	NM_000532.5:c.359_360delAT	p.Tyr120Cysfs*40	Frameshift	Early	Likely Pathogenic	NA	NA	[7]	Recurrent infections, diarrhea, metabolic acidosis, and generalized tonic–clonic seizures	Low isoleucine, methionine, threonine and proline diet with the supplementation of l-carnitine and biotin	Moderate intellectual disability and development delay
	Intron13	NM_000532.5:c.1398 + 1G > A	NA	Splice site mutation		Likely Pathogenic	35	20				
28	Exon13	NM_000532.5:C.1381G > C	p.Ala461Pro	Missense	Early	Likely Pathogenic	30	20	[66]	Vomiting	Special milk powder and oral administration of l-carnitine from 100 to 200 mg/kg every day	NA
	Exon13	NM_000532.5:c.1301C > T	p.Ala434Val	Missense		Pathogenic	32	20				
29	Exon13	NM_000532.5:C.1381G > C	p.Ala461Pro	Missense	Early	Likely Pathogenic	30	20		Poor feeding	NA	Died due to severe multiple organ failure
	Exon13	NM_000532.5:c.1301C > T	p.Ala434Val	Missense		Pathogenic	32	20				
30	Exon15	NM_000532.5:c.1535G > A	p.Arg512His	Missense	Early	Pathogenic	31	20		Poor feeding, lethargy, coma, hypotonia, metabolic acidosis and hyperammonemia	NA	Died from a severe multiple organ failure after the discharge requested by the parents at twenty-four days old
	Exon15	NM_000532.5:c.1535G > A	p.Arg512His	Missense		Pathogenic	31	20				
31	Exon11	NM_000532.5:c.1131dup	p.Val378Cysfs*5	Frameshift	Early	Likely Pathogenic	NA	NA	[119]	Lethargy, irregular breathing, with groaning and snoring, hypotonia, non-rosy skin, hypoglycemia, and metabolic acidosis	NA	Died due to respiratory failure
	Exon1	NM_000532.5:c.-10_183 + 10del203	NA	Deletion mutation		NA	NA	NA				
32	Exon11	NM_000532.5:c.1131dup	p.Val378Cysfs*5	Frameshift	Early	Likely Pathogenic	NA	NA			NA	
	Exon1	NM_000532.5:c.-10_183 + 10del203	NA	Deletion mutation		NA	NA	NA				
33	Exon8	NM_000532.5:c.838dup	p.Leu280ProfsTer11	Frameshift	Early	Pathogenic	NA	NA		Poor feeding, hypotonia, sepsisticemia, and multiple organ failure	NA	Died at seven days old after birth
	Exon11	NM_000532.5:C.1098G > C	P.Leu366Phe	Missense		Likely Pathogenic	25.8	20				
34	Intron14	NM_000532.5:c.1498 + 1G > A	NA	Splice site mutation	Early	Likely Pathogenic	34	20		NA	NA	Died shortly after birth due to metabolic abnormalities
	Intron14	NM_000532.5:c.1498 + 1G > A	NA	Splice site mutation		Likely Pathogenic	34	20				
35	Exon2	NM_000532.5:c.224A > C	p.Asp75Ala	Missense	Newborn screening	Likely Pathogenic	29.9	20	[114]	NA	NA	NA
	Exon13	NM_000532.5:c.1339C > T	p.Leu447Phe	Missense		Uncertain Significance	25.5	20				
36	Exon13	NM_000532.5:c.1301C > T	p.Ala434Val	Missense	Early	Pathogenic	32	20	[107]	Jaundice, hyperammonemia, and metabolic acidosis	Special milk powder and supplementation of l-carnitine	Regular follow-up revealed mild development delay and intellectual disability
	Exon10	NM_000532.5:c.1087 T > C	p.Ser363Pro	Missense		Likely Pathogenic	30	20				

NA, not available; MSC, Mutation Significance Cutoff

**Fig. 2:**
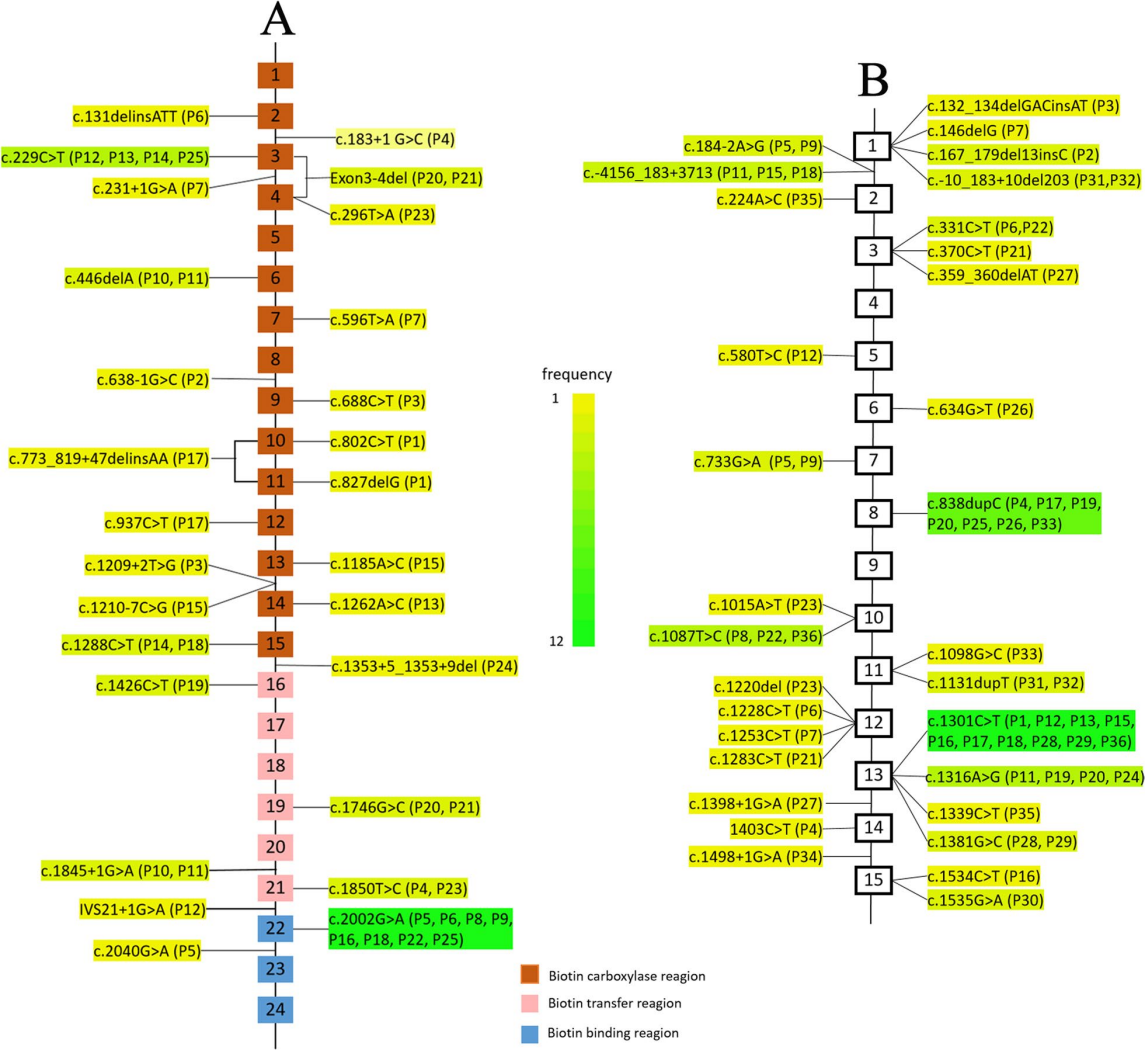
The frequency of *PCCA* and *PCCB* gene mutation types. **A** The distribution of *PCCA* variants in 25 Chinese patients diagnosed with PA. **B** The distribution of *PCCB* variants in 36 Chinese patients diagnosed with PA

The frequency of gene mutation types was calculated and shown in Fig. 2. The most common variants found in the reported Chinese PA patients were c.2002G > A in *PCCA* and c.1301C > T in *PCCB*, with frequencies of 25.0% (12/48 alleles) and 18.2% (12/66 alleles), respectively. Missense mutations has been reported to account for approximately 50% of the variations and are the most frequent type in *PCCA* and *PCCB* [54]. Our analysis also reveals that missense mutations are the most common mutant form of *PCCA* and *PCCB*, accounting for 46.0% (23/50) and 51.4% (37/72), respectively. The distribution of *PCCA* gene mutations is more dispersed in all exons except for exons 1, 5, 8, 17, 18, 20, 23, and 24, while the *PCCB* gene mutations are mainly on exons 1, 3, 12, and 13. In other countries, gene mutations of *PCCA* and *PCCB* are mostly found in exons 12, 13, 18, 19 and exons 6, 11, 12, 15, respectively [44, 55–58].

#### Genotype–phenotype correlations in Chinese PA patients

We examined reported cases of PA in Chinese patients to determine whether there are correlations between genotypes and clinical phenotypes. We identified 50 *PCCA* gene mutation sites in 25 patients (48 alleles were detected). The most common *PCCA* variant was c.2002G > A, with a frequency of 25.0% (12/48 alleles). The second most common variant was c.229C > T, accounting for 8.3% (4/48 alleles). Additionally, c.1850 T > C, c.1288C > T, c.1426C > T, c.1746G > C, exon3-4del, c.1845 + 1G > A, and c.446delA occurred at a frequency of 4.2% (2/48 alleles), while all other mutation sites were reported only once in our dataset. Homozygous variants with higher frequencies are often accompanied by a certain clinical phenotype. The c.2002G > A mutation, which results in a missense substitution of glycine by arginine at codon 668 of the *PCCA* protein (p.Gly668Arg), is one such variant. The mutant protein, which is defective in biotinylation, has been found to have only 1.4% residual activity compared to the wild-type *PCCA* protein [5]. A case study [59] reported that twin siblings with PA were homozygous for the *PCCA* c.2002G > A mutation, yet they presented slightly different clinical symptoms. The firstborn daughter developed the disease early and became more severe. She had respiratory failure, granulocytopenia, and cardiac damage with a faster disease progression. Although the second son had disorders of consciousness, feeding difficulties, hyperammonemia, and metabolic acidosis, his symptoms were relatively mild and progressed slowly. The elder daughter died after being discharged on the same day and the second son died one month after discharge. Hu et al. also reported a case of this mutation, in which the patient died at three years and nine months of age [60]. The two patients registered in Xuzhou [61] were found to have compound heterozygous mutations (*PCCA* c.2002G > A/c.2040G > A and c.2002G > A/c.131delinsATT). They exhibited growth retardation and the onset of disease started in early infancy. Zhou et al*.* [62] reported a patient with late-onset PA due to c.2002G > A homozygous mutation. This patient was admitted to hospital at the age of two years and six months for PA-associated cardiomyopathy. Despite strict dietary control and drug treatment, the child experienced several acute metabolic acidosis decompensation events and suffered severe growth and development delay which greatly affected her quality of life. Preoperative echocardiography suggested a mild left ventricular dilation with a reduced overall motion amplitude of the left ventricle and a decreased left ventricular ejection fraction. To prevent further deterioration of her cardiac, neurologic, and other systems' function or the development of irreversible complications, liver transplantation was performed. The patient had a normal function of transplanted liver and cardiac function was restored based on echocardiographic test at 13.8 months after her liver transplant. The quality of life was greatly improved, although the growth delay was not improved. A patient with c.2002G > A/c.1288C > T compound heterozygous mutation was reported by Yang et al. The patient had seizures and possible cardiomyopathy and eventually died of cardiac arrest at the age of 6.5 months [7]. These cases suggest that the clinical phenotype associated with c.2002G > A is more severe and may develop cardiomyopathy. Furthermore, c.2002G > A is one of the hot spots of *PCCA* gene mutation in China, which is consistent with the findings by Liu et al*.* [63] According to ClinVar, *PCCA* c.229C > T (p.Arg77Trp) results in a non-conservative amino acid change located in the biotin carboxylation domain and biotin carboxylase-like, N-terminal domain of the encoded protein sequence. In Taiwan, a PA patient with compound heterozygous mutations (*PCCA* c.1262A > C/c.229C > T) exhibited normal to very mild developmental delay [64]. One of 82 PA patients reported in the literature was homozygous for the *PCCA* c.229C > T mutation and exhibited convulsions and lethargy at the age of 5 years [60]. However, during the next three years, the child had no recurrence and had moderate academic performance. The height and weight of this child were in the normal range, suggesting the clinical phenotype of c.229C > T mutation is mild in these cases.

Among 36 patients with 72 *PCCB* gene mutation sites (only 66 were detected), c.1301C > T was the most common *PCCB* variant with a frequency of 18.2% (12/66 alleles), followed by c.838dupC (frequency of 12.1%, 8/66 alleles), c.1087 T > C (frequency of 6.1%, 4/66 alleles), and c.1316A > G (frequency of 6.1%, 4/66 alleles). The c.1301C > T mutation, which is located on exon13 of the *PCCB* gene, is a missense mutation (p.Ala434Val). A case of c.1301C > T homozygous mutation was reported in a child from mainland China [65], who presented with severe and early-onset clinical manifestations, including poor feeding, hyperglycinemia, hyperammonemia, metabolic acidosis, early recurrent infections, and developmental delay. Although he was positively treated such as sodium bicarbonate IV to correct acidosis, l-carnitine supplement, BCAA restriction and protein intake reduction, intellectual development was still delayed. The genotypes of 10 PA patients in Taiwan were analyzed by Chiu et al. [64]. Half of *PCCB* allele mutations reported in Taiwan are c.1301C > T(p.A434V) which is a common mutation in this region and leads to low enzyme activity, presenting the classic phenotype of PA [66]. Therefore, c.1301C > T mutation is associated with an early-onset and severe clinical symptoms.

Liu et al*.* reported a case of a child with a homozygous c.838dupC mutation in the *PCCB* gene, which caused a significant alteration in the spatial conformation of the subunit of the mutant *PCCB* protein [16]. The mutant causes the premature termination with the loss of 250 amino acids in the peptide chain of the *PCCB* protein. Despite active treatment, the child's disorders of consciousness gradually worsened, and he died of pneumonia, indicating that the c.838dupC mutation most likely contributes to severe pathogenicity. Three patients with *PCCB* c.1316 A > G mutation were diagnosed in the Jining region, Shandong province and were suspected to be a common mutation in Jining City [67]. According to Hu and others [60], one child with a c.1316 A > G homozygous mutation in the *PCCB* gene did not have another acute onset after the first onset at nine months of age. Two cases of c.1316 A > G had normal intelligence. One had an acute onset at only 20 months of age, and the other had no clinical onset. Therefore the clinical phenotype of *PCCB* c.1316A > G mutation is likely mild.

The analysis above suggests that certain gene mutations are relatively common among Chinese patients with PA, and their associated with certain phenotypes. However, for less frequent mutations, the relationship between genotype and phenotype may be less clear, possibly due to genetic heterogeneity in both *PCCA* and *PCCB* mutations. There are several challenges in correlating genotype with phenotype in patients with PA. One of these challenges is the presence of compound heterozygous mutations in many patients, which can further complicate the relationship between genotype and phenotype. In addition, determining the severity of the condition in patients with mutations in both the *PCCA* and *PCCB* genes, which encode for the PCC holoenzyme, is particularly difficult. Finally, the interaction and influence of different genes on the development and progression of the disease are not well understood [43].

### Therapeutic interventions for PA

Diet control and drug administration are common clinical treatments for PA. Organ transplantation becomes an option when the disease progresses and does not respond to diet control and drug interventions. Liver transplantation (LT) is an effective surgery since the liver can efficiently metabolize propionyl-CoA and its metabolites. Other organ transplants, such as heart and kidney [68], have also been reported when complication in these organs become severe.

PA is typically treated based on the phase of the disease that the patient is experiencing. The acute and chronic phases have different dietary and medication requirements, which are taken into consideration during treatment in the clinic. However, the primary aim of treatment for PA is to prevent protein catabolism and maintain biochemical control, growth, and development through adequate calorie intake. Long-term dietary management of PA involves restricting natural protein and supplementing with medical formulas that are enriched with Leu but free of Val, Ile, Met, and Thr to reduce the concentration of elevated metabolites [69]. Recent studies have suggested that the medical formula used for PA patients may contribute to growth retardation, despite high total protein intake. This is due to enhanced oxidation of Val and Ile in the presence of abundant Leu, known as BCAA antagonism. This imbalance makes both Val and Ile less available for anabolism, resulting in an unfavorable outcome for growth [70]. Therefore, Saleemani and colleagues have suggested that optimal protein synthesis can be achieved by reducing Leu intake and maintaining Ile and Val at the minimal level of PA recommendations. A BCAA ratio of 1:0.26:0.28 (Leu:Ile:Val) to 1:0.35:0.4 (Leu:Ile:Val) was associated with optimal protein synthesis [71]. A retrospective cohort study in Dutch evaluated both longitudinal dietary treatment and clinical course of MMA and PA patients. The study found that one-fourth of MMA and PA patients had a natural protein prescription that exceeded the recommended daily allowances (RDA). Additionally, many patients received additional amino acid mixtures (AAM) protein prescription despite already meeting the RDA for natural protein. In patients with early-onset PA, a higher natural protein prescription was associated with more frequent AMD. Therefore, it is recommended to exercise caution when prescribing AAM and to reduce protein prescriptions in patients, especially for those severely affected, who have already been given protein above RDA [72].

The current drug development for PA primarily focuses on the harmful biomarkers of the disease. l-carnitine supplement is another common treatment for PA patients by converting propionyl-CoA to propionylcarnitine which can be excreted through urine. Coenzmy Q10, an antioxidative nutritional supplement, may help prevent the chronic complications associated with mitochondrial dysfunction in PA. In a prospective study, seven patients with PA received supplements of CoQ10 in the form of ubiquinol (10 mg/kg/day for 6 months). Supplementation with ubiquinol normalized plasma CoQ10 concentrations in six patients who had shown a reduction. Furthermore, urinary citrate levels markedly increased, along with an elevation in the citrate/methylcitrate ratio [73]. Antibiotics such as metronidazole (MTZ) are often prescribed to patients with PA to inhibit the production of propionate by the intestinal microbiome. However, two patients with severe neonatal onset PA who were on chronic MTZ therapy developed axonal peripheral neuropathy. As a result, their peripheral nerve function was closely monitored during the course of MTZ treatment [74]. Arginine and sodium benzoate are used to reduce ammonia [75]. N-carbamylglutamate (NCG) has been found to be effective not only in the acute phase, but also significantly reduce ammonia levels during the chronic phase. However, additional research is needed to determine the optimal dosage of NCG [76]. Clinical studies have shown that carglumic acid is effective in reducing plasma ammonia levels in patients with PA and mitigating the frequency of hyperammonemia episodes. Additionally, carglumic acid is well-tolerated in long-term treatment [77]. Recent studies have demonstrated that a novel small molecule, HST5040 (2,2-dimethylbutyric acid), can reduce the ratio of C3/C2 and propionyl-CoA in primary hepatocytes of patients with PA in a dose-dependent manner by redistributing free and conjugated CoA pools. These findings suggest that HST5040 may be a promising drug candidate for the treatment of PA [78]. Furthermore, two allosteric pantothenate kinase activators, PZ-3022 and BBP-671, have been found to improve mitochondrial function in a mouse model of PA by improving intracellular C3:C2-CoA and plasma C3:C2-carnitine ratios and restoring liver CoA pool and acteyl-CoA to wild-type amounts in males and females [79, 80].

Liver transplantation (LT) is currently the most common surgical intervention for PA. The enzymatic activity of PCC can be restored by implanting a normal liver, which improves the metabolic stability of PA [81]. The study by Barshes et al. [82] showed that the survival rate of 12 patients with PA who received LT reached 72.2% within one year. Therefore, LT can be considered for PA patient whose condition exacerbates even with strict dietary restrictions and other medical treatments. However, LT improves certain complications but cannot completely cure PA [83]. There is still a risk of recurrence and death after LT, and ongoing follow-up treatment remains necessary.

In China, the treatment regimen for PA follows the international guideline of PA. Patients with PA should adhere to a strict and individualized nutritional intervention and receive long-term medications such as carnitine, MTZ, and NCG. In the event of severe hyperammonemia or acidosis, prompt treatment with peritoneal or blood dialysis is necessary [84]. LT becomes the first option when metabolic decompensation occurs frequently even after strict dietary restriction and drug therapy [63]. In a study by Zeng et al*.*, six Chinese patients with PA underwent living donor liver transplantation (LDLT) due to frequent metabolic decompensation and one patient with PA underwent LDLT for prophylactic treatment. All the recipients were alive with 100% allograft survival. This study also demonstrated that hepatic expressions of *PCCA* and *PCCB* was consistent at the protein level in both heterozygous donor and the healthy donor for the first time. Liver supply from relatives carrying heterozygous genes is relatively easy to obtain and less burdensome for patients’ families [85] (Additional file 1). For children with mild PA, prophylaxis is recommended for patients under one year of age [86]. It is undeniable that patients with PA may still develop complications even after LT, such as renal failure, hepatic artery thrombosis [86, 87]. However, LT can largely prevent metabolic decompensation, achieve protein diet liberalization, improve neurodevelopmental delay to some extent, and may even treat cardiomyopathy [85].

It is worth noting that extrahepatic tissues lacking PCC continue to produce toxic metabolites after LT, which means that LT cannot provide a complete cure for PA. At a tertiary center, a total of 14 children underwent LT, and three of them died after LT. Among the 11 survivors, two experienced metabolic stroke but made a full recovery, and three developed mild cardiomyopathy after LT [88]. In some cases, PA complications may continue to progress even after a successful LT. For example, there was a case, who presented with a fatal metabolic stroke 11 years after undergoing a successful LT [89]. Two case were reported to develop recurrent cardiomyopathy following LT [90]. Several issues need to be addressed when considering LT for patients with PA: (1) minimizing LT complications such as rejection, hepatic artery thrombosis, cytomegalovirus/Epstein-Barr virus infection, and biliary complications; (2) determining the optimal timing for LT, and considering prophylactic LT; and (3) assessing long-term safety and feasibility of protein intake liberalization after LT.

### Emerging therapies

Hepatocyte transplantation and hepatic progenitor cell transplantation have also been investigated as emerging treatments for urea cycle defects, including PA, in clinical trials [91, 92]. Gene therapy is emerging as a promising therapeutic approach for patients with PA, although it still faces challenges [93]. The first gene therapy product, AAV9-hPCCA (NCATS-BL0746), using adeno-associated virus serotype 9 to deliver human *PCCA*, was granted a rare pediatric disease designation by the U.S. Food and Drug Administration (FDA) for clinical trials on *PCCA*-related PA [94].

Recently, an enzyme replacement approach to treat PA has been reported. This approach involves the administration of a combination of two mRNAs encoding human *PCCA* and *PCCB* encapsulated in biodegradable lipid nanoparticles (LNPs), to a murine model with a hypomorphic phenotype, Pcca(− / −)(p.A138T). The treatment resulted in the production of functional PCC enzyme in the liver and a reduction in primary disease-associated toxins in a dose-dependent manner in both 3- and 6-month repeat-dose studies in mice with PA [95].

## Conclusion

PA is a genetic disease with a poor prognosis that requires early detection and treatment for efficient preventive management. LC–MS/MS and GC–MS are essential in early clinical screening and diagnosis. More attention should be paid to the specific effects of *PCCA* and *PCCB* mutations on the organ complications for the precise treatment strategy. Common mutations in China, such as *PCCA* c.2002G > A and *PCCB* c.1301C > T, require further clinical studies, as well as ancestry analysis and haplotype mapping, to determine if they are hot spot mutations in the overall Chinese population. However, we would like to note that the availability of data for ancestry analysis and haplotype mapping for these variants is currently unknown, and as such, we cannot determine if they are hot spot mutations in the Chinese population. The mainstay of nutrition therapy is a low protein intake while ensuring essential requirements of amino acids, Ile, Val, Met, and Thr. Medical formula is recommended to supplement the RDA not met by natural protein, and plasma amino acid levels should be monitored closely. Further research is needed to determine the optimal BCAA ratio in patients with PA to optimize their total protein synthesis. Selection of therapeutic drugs for hyperammonemia should also be carefully examined due to different mechanisms of hyperammonemia occurring in PA. Although patients with PA have been reported in most regions of China, neonatal screening for PA is mainly concentrated in certain areas, which limits the research results' selection and bias. Therefore, obtaining accurate information and data nationwide based on the application of neonatal screening strategies will provide a scientific basis for the treatment of PA.

### Supplementary Information

**Additional file 1**. Demographics, preoperative characteristics, and operative findings of PA patients who underwent liver transplant.

## Data Availability

The datasets used and/or analyzed during the current study can be obtained from the corresponding author upon reasonable request.

## Abbreviations

PA: Propionic acidemia
propionyl-CoA: Propionyl coenzyme A
PCC: Propionyl carboxylase
AMD: Acute metabolic decompensations
BCAA: Branched-chain amino acid
LC–MS/MS: Liquid chromatography-tandem mass spectrometry
GC/MS: Gas chromatography-mass spectrometry
2MCA: 2-Methylcitric acid
C3: Propionylcarnitine
C2: Acetylcarnitine
MMA: Methylmalonic acidemia
qPCR: Quantitative polymerase chain reaction
NGS: Next generation sequencing
NCG: N-carbamylglutamate
LT: Liver transplantation
LDLT: Living donor liver transplantation
