# Supplementary material for: Prevalence of propionic acidemia in China
Source: Orphanet J Rare Dis. 2023 Sep 9;18:281. doi: 10.1186/s13023-023-02898-w (PMC10493020; doi:10.1186/s13023-023-02898-w)
Supplement: Supplementary file 1 — Additional file 1. Demographics, preoperative characteristics, and operative findings of PA patients who underwent liver transplant. [file 13023_2023_2898_MOESM1_ESM.doc]

Table S1 Demographics, preoperative characteristics, and operative findings of PA patients who underwent liver transplant

| Sex | Age at onset/ diagnosis | Genetic testing | Initial clinical manifestations | Age at LT | Indication for LT | Donor | Heterozygous donor | Posttransplant complications | Outcomes | LT selection criteria |
| --- | --- | --- | --- | --- | --- | --- | --- | --- | --- | --- |
| Female | NA/newborn screening | PCCB c.1301C>T/c.1534C>T | Poor feeding, vomiting, recurrent episodes of metabolic acidosis, hyperammonemia and moderate mental retardation | 10 months | Frequent metabolic decompensations and moderate developmental delay | Mother | NA | NA | No life-threatening metabolic acidosis during the 12-month follow-up period. | For patients with poor diet and drug control, and frequent attacks of metabolic disorders |
| Female | 10 days/8 months | PCCA c.2002G>A/c.2002G>A | Poor feeding and episodic vomiting | 2.7 years | Frequent metabolic decompensations and dilated cardiomyopathy | Mother | Yes (c.2002G>A) | Cytomegalovirus Viremia | With a normal diet and L-carnitine supplementation, the patient did not encounter any further specific complications of PA, nor did she experience any transplant-related complications during the follow-up period. |
